# Epiretinal Membrane Is Associated with Acquired Vitelliform Lesion Morphometrics in Intermediate Age-Related Macular Degeneration

**DOI:** 10.1016/j.xops.2026.101228

**Published:** 2026-05-12

**Authors:** Alberto Quarta, Rouzbeh Abbasgholizadeh, Jianfeng Huang, Shinichiro Chujo, Ceren Soylu, Sweetha Bindu Velaga, Alireza Mahmoudi, Sophiana Lindeberg, Mai Alhelaly, Muneeswar G. Nittala, Giulia Corradetti, Rodolfo Mastropasqua, Charles Wykoff, Srinivas R. Sadda

**Affiliations:** 1 Doheny Eye Institute, Pasadena, California; 2 Department of Ophthalmology, David Geffen School of Medicine, University of California, Los Angeles, California; 3 Beijing Hospital, National Center of Gerontology, Institute of Geriatric Medicine, Chinese Academy of Medical Sciences, Beijing, PR China; 4 Department of Ophthalmology, Mie University Graduate School of Medicine, Mie, Japan; 5 Ophthalmology Department, Tanta University, Tanta, Egypt; 6 Department of Neurosciences, Imaging and Clinical Sciences, University “G. d'Annunzio” Chieti-Pescara, Chieti, Italy; 7 Retina Consultants of Texas, Retina Consultants of America, Houston, Texas

**Keywords:** Age-related macular degeneration, Acquired vitelliform lesion, Epiretinal membrane

## Abstract

**Purpose:**

To investigate the impact of epiretinal membrane (ERM) on the structural characteristics of acquired vitelliform lesions (AVLs) in eyes with intermediate age-related macular degeneration (iAMD).

**Design:**

Cross-sectional study.

**Participants:**

Eyes of patients with iAMD-associated AVLs were identified from a tertiary referral center database.

**Methods:**

Multimodal imaging, including spectral-domain OCT, was reviewed. Eyes were stratified according to the presence (ERM + AVL) or absence (AVL-only) of ERM. Quantitative OCT parameters, including apical AVL height and width, and qualitative structural features such as intraretinal hyperreflective foci (IHRF), ellipsoid zone (EZ), and external limiting membrane (ELM) disruption, and AVL location relative to drusen, were assessed by masked graders. Effect sizes were calculated using Hedges' *g*. Group comparisons were performed using appropriate parametric or nonparametric tests.

**Main Outcome Measures:**

Differences in AVL structural characteristics between eyes with and without ERM.

**Results:**

A total of 234 eyes with age-related macular degeneration (AMD)-associated AVLs were included, of which 17 eyes had concomitant ERM. Eyes with ERM demonstrated significantly greater apical AVL height compared with AVL-only eyes (169.9 ± 52.5 μm vs. 134.8 ± 55.0 μm; *P* = 0.016). Acquired vitelliform lesion width was greater in the ERM + AVL group, although this difference did not reach statistical significance (811.1 ± 251.0 μm vs. 676.8 ± 416.0 μm; *P* = 0.057). The prevalence of IHRF, EZ disruption, ELM disruption, and AVL location over drusen did not differ significantly between groups. Effect size analysis demonstrated a moderate positive effect for apical AVL height (Hedges' *g* ≈ 0.65; 95% confidence interval [CI] ∼0.15 to 1.10), whereas AVL width showed a small-to-moderate effect size with CIs crossing 0.

**Conclusions:**

In eyes with AMD-associated AVLs, the presence of ERM is associated with increased AVL height without corresponding differences in other outer retinal structural features. These findings suggest that vitreomacular traction may selectively influence AVL configuration. Longitudinal studies are needed to determine whether traction-related modulation of AVL morphology impacts AMD disease progression and AVL collapse.

**Financial Disclosure(s):**

Proprietary or commercial disclosure may be found in the Footnotes and Disclosures at the end of this article.

Age-related macular degeneration (AMD) includes a heterogeneous spectrum of degenerative changes affecting the macula, ranging from early drusen formation to advanced atrophy and macular neovascularization (MNV).^1^^,^^2^ Within this spectrum, acquired vitelliform lesions (AVLs) have been increasingly recognized as a distinct phenotype, characterized by subretinal accumulation of hyperreflective material. Acquired vitelliform lesions in AMD have been associated with an increased risk of progression to geographic atrophy (GA) and MNV, suggesting that they may represent a marker of retinal pigment epithelium (RPE) dysfunction and disease activity rather than an epiphenomenon.^3^, ^4^, ^5^, ^6^

Another age-associated change involves progressive weakening of adhesion at the vitreoretinal interface between the posterior cortical vitreous and the inner limiting membrane, with future epiretinal membrane (ERM) development.^7^ Epiretinal membrane is a common vitreomacular interface disorder characterized by fibrocellular proliferation on the inner retinal surface, frequently associated with aging and posterior vitreous detachment.^7^, ^8^, ^9^ Through tangential and anteroposterior traction, ERM can induce progressive distortion of the foveal architecture including abnormalities of the foveal bouquet, leading to disruption of outer retinal layers and secondary photoreceptor dysfunction.^10^^,^^11^ Chronic tractional forces have been implicated in the development of pseudovitelliform or vitelliform-like material accumulation presumably related to impaired outer retinal metabolism and altered RPE–photoreceptor interactions.^10^^,^^12^

As both ERM and AMD share a strong association with aging, unsurprisingly, their coexistence is frequently observed in clinical practice.^13^, ^14^, ^15^ However, despite growing recognition of traction-related outer retinal changes and the prognostic implications of AVLs in AMD, no studies to date have systematically evaluated the potential impact of ERM on AVL morphology, behavior, or associated disease progression in AMD eyes. Specifically, it remains unclear whether vitreomacular traction exerted by ERM may influence AVL characteristics, potentially modifying their structural features or clinical course.

The aim of this study was to investigate the impact of ERM in eyes with AMD-associated AVL. We compared clinical and multimodal imaging characteristics between AMD eyes with coexisting ERM and AVL and those with AVL alone to assess whether the presence of ERM is associated with distinct AVL features or may represent a modifying factor in this AMD phenotype.

## Methods

### Study Design and Participants

This cross-sectional multicenter analysis was conducted using imaging and clinical data derived primarily from the Retina Consultants of Texas database, supplemented by additional cases obtained from the Doheny Eye Center at the University of California, Los Angeles (Pasadena, CA). The study adhered to the principles of the Declaration of Helsinki, complied with the Health Insurance Portability and Accountability Act, and was approved by the Institutional Review Board of the University of California, Los Angeles. Informed consent was waived due to the retrospective design of the study.

Medical records and multimodal retinal imaging of patients diagnosed with intermediate AMD (iAMD) and foveal AVLs with or without ERM were reviewed according to previous definitions.^4^^,^^5^ Eyes were included if they met the following criteria: (1) diagnosis of iAMD according to Beckman criteria as the presence of extensive medium drusen (63–125 μm) or ≥1 large druse (>125 μm), with or without pigmentary abnormalities, and without features of late AMD (GA or neovascular AMD);^16^ (2) availability of baseline volume OCT scans (20 × 20 degrees; 1024 × 49 with automatic real-time tracking >9 centered on the fovea); and (3) diagnosis of idiopathic ERM confirmed by clinical examination and OCT.^8^ The exclusion criteria included (1) secondary ERM (e.g., associated with retinal vascular disease, uveitis, trauma, retinal detachment, laser retinopexy, or prior retinal surgery), coexisting macular disease affecting the retinal architecture (such as neovascular AMD defined by Consensus on Neovascular Age-Related Macular Degeneration Nomenclature criteria^17^); (2) OCT images with significant motion artifacts or segmentation errors precluding reliable analysis; (3) retinopathies other than AMD; and (4) presence of incomplete/complete RPE and outer retinal atrophy according to Classification of Atrophy Meeting criteria.^18^ Eyes with a concomitant ERM constituted the ERM + AVL group, while eyes with AVL in the absence of ERM served as controls (AVL-only group).

### Study Population and Procedures

All subjects underwent a comprehensive ophthalmic evaluation, including detailed medical history collection, best-corrected visual acuity assessment, intraocular pressure measurement, slit lamp biomicroscopy, and spectral-domain OCT (Spectralis). When available, ultra-widefield fundus photography (Optos California; Optos), confocal scanning laser ophthalmoscopy multicolor, and fundus autofluorescence (Spectralis, Heidelberg Engineering GmbH) were reviewed. Multimodal imaging was reviewed by 2 masked and independent graders (A.Q. and R.A.) with further confirmation by a third senior investigator (S.R.S.) with regards to the presence of ERM, AMD, and AVL.

### Structural OCT Assessment

**Figure 1:**
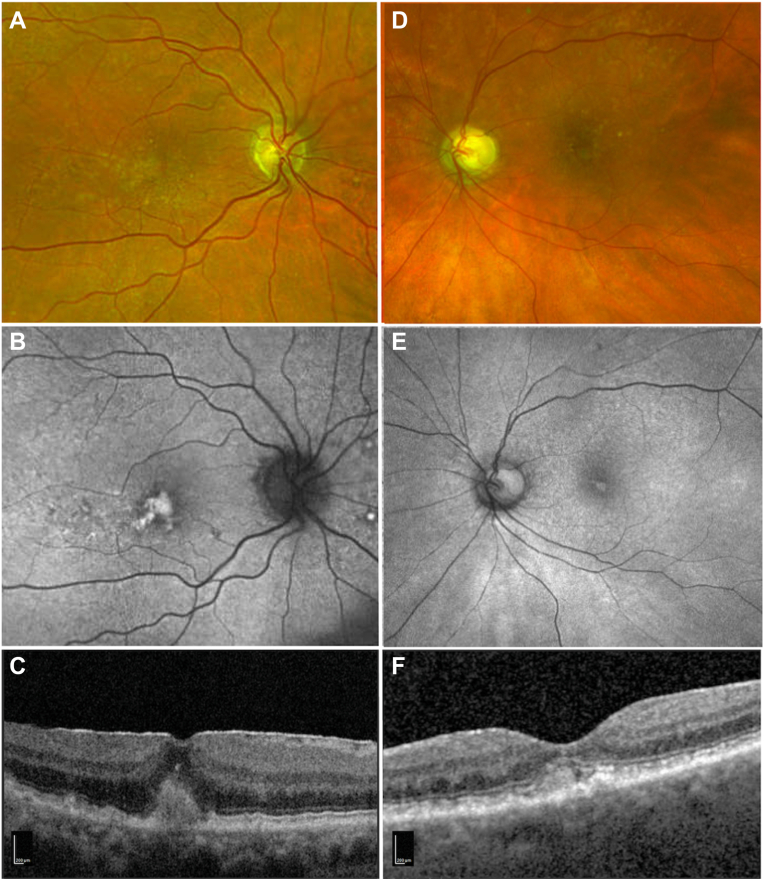
Multimodal imaging of AVLs with and without ERM. Representative multimodal images of eyes with intermediate AMD and AVLs. **A–C,** Eye with AVL and concomitant ERM. **A,** Color fundus photograph demonstrating macular pigmentary alteration and subtle vitreoretinal interface distortion. **B,** Fundus autofluorescence image showing a focal area of increased autofluorescence corresponding to the AVL. **C,** Spectral-domain OCT B-scan illustrating a dome-shaped accumulation of hyperreflective subretinal material consistent with an AVL, with associated inner retinal surface irregularity and distortion of the foveal contour consistent with ERM. **D–F,** Eye with AVL without ERM. **D,** Color fundus photograph demonstrating intermediate AMD features without evident vitreoretinal interface abnormality. **E,** Fundus autofluorescence image showing focal hyperautofluorescence corresponding to the AVL. **F,** Spectral-domain OCT B-scan demonstrating a dome-shaped subretinal hyperreflective lesion consistent with an AVL, in the absence of significant inner retinal distortion. Scale bars = 200 μm. AMD = age-related macular degeneration; AVL = acquired vitelliform lesion; ERM = epiretinal membrane.

Qualitative and quantitative OCT features were evaluated by 2 independent masked graders experienced in retinal imaging (A.Q. and R.A.), and any discrepancies were resolved through subsequent discussion. Foveal AVL were identified on spectral-domain OCT as dome-shaped accumulations of hyperreflective material located in the subretinal space, bordered posteriorly by the inner surface of the RPE/Bruch's membrane complex and anteriorly by the photoreceptor layers, including the ellipsoid zone (EZ), external limiting membrane (ELM), or in cases of advanced overlying retinal alteration, the outer portion of the outer nuclear layer^4^^,^^5^^,^^19^ (Fig 1). When available, fundus autofluorescence was reviewed to confirm corresponding areas of hyperautofluorescence. Associated structural OCT features were graded according to previously published criteria from Classification of Atrophy Meeting Report 5.^19^ These features included the presence of intraretinal hyperreflective foci (IHRF), and attenuation or disruption of the EZ or ELM. Intraretinal hyperreflective foci were defined as punctate intraretinal lesions measuring ≥3 pixels in diameter with reflectivity equal to or greater than that of the RPE.^20^ In cases in which fundus autofluorescence images were unavailable, lesions were classified as AVLs only if they were isolated and demonstrated a minimum apical height of 45 μm to be distinguished from subretinal drusenoid deposit on OCT, consistent with previous works.^21^^,^^22^ Disruption or attenuation of the ELM and EZ was assessed based on focal discontinuity or reduced reflectivity of the respective bands on OCT. Quantitative assessments included AVL apical height and basal width, and subfoveal choroidal thickness below the AVL center.^4^ Measurements of subfoveal choroidal thickness associated to AVL were obtained using the built-in caliper tool on the relevant B-scan in the Spectralis HEYEX software. Choroidal thickness was measured as the vertical distance between the posterior border of the RPE/Bruch's membrane complex and the inner surface of the sclera. Acquired vitelliform lesion apical height was defined as the distance from the apex of the lesion to the underlying RPE; in cases in which the RPE was attenuated or indistinct, the measurement was extended to the Bruch's membrane.^21^ The basal width of the AVL was defined as the horizontal distance between the lateral margins of the lesion measured along the plane of the RPE layer.^21^ The grading approach has been previously reported and demonstrated excellent intergrader reproducibility with intraclass correlation coefficients >0.90.^3^ In eyes with ERM, the membrane was defined as a hyperreflective line internal to the retinal surface but maintaining points of contact with the inner retina and its stage was assessed as previously described.^8^

### Statistical Analysis

Continuous variables were reported as mean ± standard deviation or median with interquartile range as appropriate, and categorical variables as frequencies and percentages. Variables were assessed for normality distribution using the Shapiro–Wilk test. Comparisons between groups were conducted using independent-sample *t* tests or Mann–Whitney *U* tests for continuous variables and chi-square or Fisher exact tests for categorical variables. Effect sizes were calculated using Hedges' g with corresponding 95% confidence intervals (CIs). Hedges' g effect sizes were interpreted using standard thresholds (0.2 = small, 0.5 = medium, 0.8 = large). A 2-tailed *P* value <0.05 was considered statistically significant. All analyses were performed using R version 4.1.0 (R Foundation for Statistical Computing).

## Results

### Study Population and Demographics

A total of 234 eyes from 180 patients with AMD-associated AVLs were included in the analysis; 17 eyes from 12 patients presented with a concomitant ERM (ERM + AVL group), while 217 eyes from 168 patients had AVL in the absence of ERM (AVL-only group). The proportion of female patients was comparable between groups (ERM + AVL: 58.3%; AVL-only: 77%; *P* = 0.914). The mean age for the ERM + AVL group was 81.3 ± 6.7 years and 73.88 ± 9.41 years for the AVL-only group.

### Comparison of AVL Structural Characteristics

**Figure 2:**
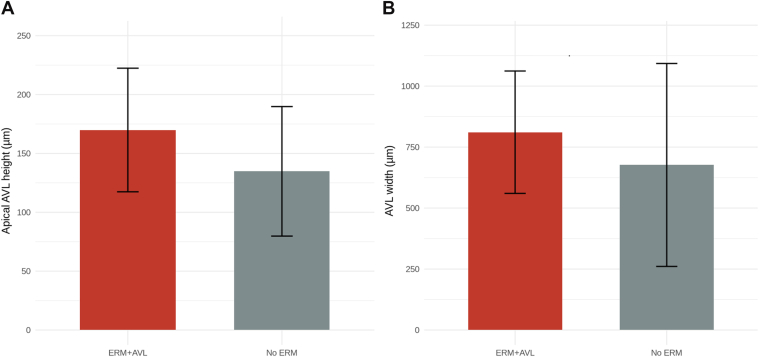
Comparison of AVL structural metrics by ERM status. **A,** Bar plot showing the mean apical AVL height (μm) for the 2 groups. Eyes with ERM + AVL exhibit a higher mean apical AVL height compared with the no-ERM group. Error bars represent variability (± standard deviation), indicating moderate spread in both groups, with slightly greater variability in the no-ERM group. **B,** Bar plot showing the mean AVL width (μm). Similarly, the ERM + AVL group demonstrates a greater mean AVL width than the no-ERM group. AVL = acquired vitelliform lesion; ERM = epiretinal membrane.

Eyes with ERM demonstrated a significantly greater apical AVL height compared with eyes without ERM (169.9 ± 52.5 μm vs. 134.8 ± 55.0 μm; *P* = 0.016). Choroidal thickness was 256.8 ± 88.0 μm in eyes without ERM and 185.47 ± 87.39 μm in eyes with ERM (*P* = 0.060). Acquired vitelliform lesion width also tended to be larger in the ERM + AVL group (811.1 ± 251.0 μm vs. 676.8 ± 416.0 μm), although this difference did not reach statistical significance (*P* = 0.057) (Fig 2). The prevalence of IHRF overlying the AVL was similar between groups (ERM + AVL: 23.5% vs. AVL-only: 34.0%; *P* = 0.327). Likewise, disruption of the EZ and ELM did not differ significantly between eyes with and without ERM (EZ disruption: 76.5% vs. 89.9%, *P* = 0.202; ELM disruption: 64.7% vs. 68.7%, *P* = 0.713). The proportion of AVLs located over drusen was numerically higher in the ERM + AVL group (47.1%) compared with the AVL-only group (31.0%), although this difference was not statistically significant (*P* = 0.196) (Table 1). Among eyes with ERM (n = 17), stage distribution was as follows: stage 1 in 58.8% (n = 10), stage 2 in 29.4% (n = 5), and stage 3 in 11.8% (n = 2) of cases.

**Table 1:** Structural and Demographic Characteristics of Eyes with AVLs with and without ERM

Variable	ERM	No ERM	*P* Value
Apical AVL height	169.90 ± 52.50	134.80 ± 55.00	**0.016**∗
AVL width	811.10 ± 251.00	676.80 ± 416.00	0.057∗
IHRF over AVL			0.327†
Yes	4/17 (23.5%)	74/217 (34.0%)	
No	13/17 (76.5%)	143/217 (66.0%)	
EZ disruption			0.202†
Yes	13/17 (76.5%)	195/217 (89.9%)	
No	4/17 (23.5%)	22/217 (10.1%)	
ELM disruption			0.713†
Yes	11/17 (64.7%)	149/217 (68.7%)	
No	6/17 (35.3%)	68/217 (31.3%)	
Gender			0.914†
Female	7/12 (58.3%)	130/168 (77%)	
Male	5/12 (41.7%)	87/168 (51.7%)	
AVL over drusen			0.196†
Yes	8/17 (47.1%)	68/217 (31.0%)	
No	9/17 (52.9%)	149/217 (69.0%)	

AVL = acquired vitelliform lesion; ERM = epiretinal membrane; ELM = external limiting membrane; EZ = ellipsoid zone; IHRF = intraretinal hyperreflective foci.

Statistically significant *P* value is reported in bold.

∗ Mann–Whitney *U* tests.

† Fisher exact tests.

### Effect Size and CI Analysis

Effect sizes were calculated using Hedges' *g* to quantify the magnitude of differences between groups. Apical AVL height demonstrated a Hedges' *g* of approximately 0.65, (95% CI 0.15–1.10). Acquired vitelliform lesion width showed a Hedges' *g* of approximately 0.30–0.35 (CI 95% –0.05 to 0.75). All other evaluated structural parameters demonstrated small effect sizes (*g* < |0.20|), with CIs including zero.

## Discussion

In this study, we evaluated the structural characteristics of AVL in eyes with iAMD according to the presence or absence of ERM. Eyes with coexisting ERM demonstrated a significantly greater apical AVL height compared with eyes with AVL alone, while AVL width showed a nonsignificant trend toward larger dimensions in the ERM group. Other outer retinal features including IHRF were similar between groups. Effect size analysis further confirmed that differences between groups were primarily driven by an increased AVL height in eyes with ERM.

Acquired vitelliform lesion is increasingly recognized as part of the heterogeneous phenotypic spectrum of iAMD and these lesions have been associated with an increased risk of progression to both GA and MNV.^3^^,^^5^ Prior studies have suggested that AVLs may reflect a state of RPE dysfunction and impaired outer retinal metabolism, rather than a static accumulation of subretinal material.^3^^,^^21^ The structural evolution of AVLs including changes in size, reflectivity, and associated photoreceptor alterations has therefore gained interest as a potential biomarker of disease activity and progression to GA.^12^

Vitreoretinal disorders are gaining importance in patients with AMD because it seems that they are associated with higher risk of atrophy progression and MNV.^23^, ^24^, ^25^, ^26^ Epiretinal membrane represents a common age-related vitreomacular interface abnormality and is known to induce tangential and anteroposterior tractional forces on the epiretinal surface.^10^^,^^15^^,^^27^ The concept that inner retinal traction can influence outer retinal integrity is supported by prior OCT-based investigations. Cho et al^28^ demonstrated a significant correlation between inner retinal deformation and outer retinal layer disruption in eyes with idiopathic ERM, suggesting that tractional forces at the vitreoretinal interface may propagate through the retinal layers and contribute to photoreceptor and RPE alteration. Similarly, prognostic studies in ERM surgery including the work of Kauffmann et al^29^ have highlighted the importance of preoperative outer retinal biomarkers in determining outcomes, reinforcing the close structural interdependence between inner retinal changes and outer retina. Tractional forces can lead to distortion of the foveal architecture and have been implicated in changes of the foveal bouquet including displacement and disruption of cone outer segments.^10^ Prior work has demonstrated that chronic traction at the vitreoretinal interface may result in secondary outer retinal changes, including pseudovitelliform or vitelliform-like material accumulation likely mediated by altered photoreceptor–RPE interactions and impaired outer segment turnover.^30^^,^^31^ Although ERM originates at the vitreoretinal interface, tractional forces are known to propagate across retinal layers, particularly at the fovea, where retinal architecture is uniquely specialized and mechanically vulnerable.^8^^,^^32^ This mechanism is consistent with our findings in which eyes with ERM exhibited increased apical AVL height without a corresponding increase in markers of generalized outer retinal damage.

Importantly, the absence of significant differences in EZ, ELM, and IHRF between groups suggests that traction may preferentially modulate AVL morphology rather than accelerate the degenerative processes per se. This supports the notion that ERM may act as a biomechanical modifier of existing AMD-related changes, influencing the spatial configuration of subretinal material rather than its composition. These observations raise clinically relevant questions regarding the interpretation of AVL morphology in eyes with coexisting vitreomacular interface abnormalities. Increased AVL height in the setting of ERM may reflect traction-related remodeling rather than intrinsic progression of AMD. This distinction is particularly important given emerging evidence that AVL morphometric characteristics are not merely descriptive but prognostically meaningful. Mahmoudi et al^5^ demonstrated that AVLs exhibit heterogeneous longitudinal behavior, with some lesions regressing while others collapse and progress to macular atrophy, and identified AVL height as one of the strongest predictors of subsequent atrophy development.^3^ Within this framework, our finding that ERM is associated with increased AVL height raises the possibility that vitreomacular traction may artificially augment this morphometric biomarker, potentially confounding risk stratification based on AVL dimensions alone. Accordingly, traction-modified AVL morphology may not carry the same prognostic implications as height increases driven by intrinsic outer retinal and RPE degeneration. Longitudinal studies incorporating serial OCT and OCT angiography will therefore be essential to determine whether ERM-associated increases in AVL height confer differential risk for subsequent atrophy enlargement or MNV, or whether they represent a partially reversible structural phenotype. Notably, our data suggest that vitreomacular interface abnormalities may selectively influence the vertical configuration of vitelliform material without necessarily exacerbating generalized outer retinal disruption, further supporting the concept of a biomechanical contribution distinct from degenerative progression.

Importantly, emerging evidence suggests that specific AMD-associated deposits may further modulate the anatomic expression and clinical implications of ERM. Wilde et al demonstrated that the presence of subretinal drusenoid deposit significantly influences both baseline OCT characteristics and surgical outcomes in eyes with ERM, indicating that the underlying outer retinal and subretinal environment shapes the manifestation of vitreomacular interface disease.^33^ Observational studies of eyes with drusen and ERM after vitrectomy similarly support an interaction between tractional forces and AMD-related structural substrates.^34^ Overall, the presence of subretinal drusenoid deposits has been associated with poorer anatomical and functional outcomes after ERM surgery, likely reflecting more advanced outer retinal dysfunction, while eyes with coexisting drusen and dry AMD may demonstrate reduced visual recovery after vitrectomy compared with eyes without AMD-related deposits.

Our findings suggest that while increased AVL height remains a relevant biomarker of progression risk, its interpretation should be contextualized by vitreomacular interface status, as ERM-related traction may artificially augment lesion height independent of underlying degenerative processes.

Several limitations should be considered. The retrospective design and relatively small number of eyes with ERM limit the possibility to draw definitive conclusions regarding causality or disease progression. Furthermore, as the OCT acquisition protocols were not specifically optimized for viewing vitreous–retina relationships, we could not fully assess for the presence of subtle vitreomacular traction, either directly on the ERM or to adjacent regions of retina. In addition, the cross-sectional nature of the analysis precludes assessment of temporal relationships between ERM development, AVL evolution, and downstream outcomes such as atrophy enlargement or MNV. As such, the reported associations represent an explorative, unadjusted, eye-level comparisons and should be interpreted cautiously. Nevertheless, the observed effect size was moderate and consistent across eyes, supporting the biological plausibility of the findings. Future studies should consider potential intereye factors in modeling the potential influence of ERM on AVL lifecycle. The current study should be seen as an initial hypothesis-generating investigation, and longitudinal studies incorporating serial multimodal imaging are needed to clarify whether traction-related modulation of AVL morphology influences the natural history of AMD, including the risk of progression to advanced disease stages. Such investigations may also help determine whether the presence of ERM should be considered when interpreting AVL-related biomarkers or when stratifying risk in AMD eyes.

In conclusion, ERM was associated with increased AVL height in eyes with iAMD without corresponding differences in other outer retinal OCT features. These results suggest that vitreomacular interface status may influence AVL configuration and should be considered when interpreting this phenotype. Prospective longitudinal studies are needed to clarify the prognostic significance of these observations.
